# Characterisation of larval habitats, species composition and factors associated with the seasonal abundance of mosquito fauna in Gezira, Sudan

**DOI:** 10.1186/s40249-017-0242-1

**Published:** 2017-02-08

**Authors:** Mostafa M. Mahgoub, Eliningaya J. Kweka, Yousif E. Himeidan

**Affiliations:** 1 Faculty of Science, Imam Muhammad Bin Saudi Islamic University, Al-Riyadh, Saudi Arabia; 2 Tropical Pesticides Research Institute, Division of Livestock and Human Diseases Vector Control, Mosquito Section, P.O. Box 3024, Arusha, Tanzania; 3 Department of Medical Parasitology and Entomology, Catholic University of Health and Allied Sciences, P.O. Box 1464, Mwanza, Tanzania; 4 Vector Control Unit, Africa Technical Research Centre, P.O. Box 15500, Arusha, Tanzania

**Keywords:** *Anopheles*, *Culex*, *Aedes*, Larvae, Habitats, Seasonal abundance, Mosquitoes, Gezira

## Abstract

**Background:**

Larval source management (LSM), which requires an understanding of the ecology and composition of the local mosquito fauna, is an important parameter in successful vector control programmes. The present study was conducted to understand the distribution of larval habitats, species composition and factors associated with the seasonal abundance of mosquito larvae in Gezira irrigation Scheme in Gezira state, central Sudan.

**Methods:**

Cross-sectional larval surveys were carried out in the communities of Barakat (urban) and El-Kareiba (semi-urban), in Wad Madani, Gezira. A standard dipper was used for sampling larvae in all possible breeding sites and enamel bowls were employed for larvae sorting. Habitats were characterised using physical features and all larvae specimens were identified morphologically.

**Results:**

A total of 331 larval habitats were surveyed, out of which 166 were found to be positive breeding sites for *Anopheles* (56.78%), Culicinae (29.67%) and *Aedes* (13.55%) species. A total of 5 525 larvae collected were categorised as *Culex* (2 617, 47.37%), *Anopheles* (2 600, 47.06%) and *Aedes* (308, 5.57%). There was a high number of positive habitats during the rainy season, while the lowest proportion was reported during the hot dry season, in both study sites (Barakat [*χ* ^2^ = 10.641, *P* = 0.0090], El-Kareiba [*χ* ^2^ = 23.765, *P* = 0.0001]). The main breeding site for *Anopheles* larvae was leaking water pipes (51.5%), followed by irrigation channels (34.2%), hoof prints (6.4%), tyre tracks (5.5%) and water tanks (2.4%). A logistic regression analysis showed that the abundance of *Anopheles* larvae was reduced by the presence of predators (backswimmers, tadpoles) and grass cover. Adult productivity (number of adult females emerged/m^2^) was not homogeneousfor all habitats; the highest productivity was found in irrigation channels (0.78 females/m^2^) for *Anopheles*, and in septic tanks (2.86 females/m^2^) for Culicinae and (0.86 females/m^2^) for *Aedes. Anopheles arabiensis* was found to be the dominant *Anopheles* species. This study documented the presence of *An. funestus* in central Sudan for the first time.

**Conclusions:**

Maintaining leaking water pipes and adopting intermittent irrigation are recommended for LSM, as these surveyed habitats represent the main source of maintaining the local mosquito population during the hot dry season.

**Electronic supplementary material:**

The online version of this article (doi:10.1186/s40249-017-0242-1) contains supplementary material, which is available to authorized users.

## Multilingual abstracts

Please see Additional file 1 for translation of the abstract into the five official working languages of the United Nations.

## Background

Mosquito-borne diseases are becoming a serious global burden. Climate changes due to global warming are leading to the spread of disease vectors and pathogens in formerly disease-free areas [1–4]. These changes affect the seasonality of vectors and, subsequently, the distribution and transmission patterns of diseases [1, 2, 5, 6].

Malaria is an important mosquito-borne disease in Sudan. Sudan is one of the countries with a high malaria burden in Sub-Saharan Africa [7]. Many recent outbreaks of mosquito-borne diseases have occurred in Gezira state, including yellow fever in 2005, and Rift Valley fever in 2007 and 2010, and malaria always occurs during and after the rainy season [3, 4, 6, 7]. In the recent, Larva; source management has gained a wide attention as it is throughout to be most useful target. It deals with a developmental stage that cannot move outside the targeted site. Hence, thought to be useful if well planed.

In Sudan, irrigated areas cover around 1.5% of the total farmland, providing suitable and stable mosquito breeding sites. Mosquito species distribution is associated with climatic zones and degrees of aridity [8, 9]. In the dry savannah areas of central and eastern Sudan, very rare larval habitats are found during the dry season [8–11]. Gezira is located in central Sudan, along the Blue Nile river, between the latitude 13–15.2 °N and longitude 32.5–34 °E. Huge ecological changes have occurred after Gezira Scheme such as increased irrigated areas, deforestation and hence became one of the largest irrigation projects in the world to became operational. It started in 1925 with 300 000 feddans, due to expansion of land size, nowadays covers an area of some 2 million feddans (one feddan = 0.42 ha) principally under gravity irrigation [12].

The current mosquito species list in Sudan is based on surveys carried out by Lewis in the mid-1950s, not taking into account the environmental and climate changes that might have influenced the re-distribution of vector species [13]. Land use patterns and climate changes are factors considered to be the drivers of species composition and dynamics [14–16]. The expansion of Gezira scheme might have influenced changes in species composition in this state [7]. Four more species of mosquitoes, *Anopheles sergenti*, *An. domicolus*, *An. seydeli* and *An. brohieri*, have been identified by studies conducted by Gillies and De Meillon [17]. In 1997, studies by Nugud et al. identified 31 *Anopheles* species, including two more new species [18]. These variations show that there is a change of species composition in space and time, and have raised a need for re-assessing mosquito species and composition in different areas in order to be able to better select and implement vector control and intervention tools.

The aim of the current study was to assess the factors associated with seasonal abundance of larval species and characterise larval habitats for mosquito fauna in the Gezira Scheme in central Sudan.

## Methods

### Study sites

**Fig. 1:**
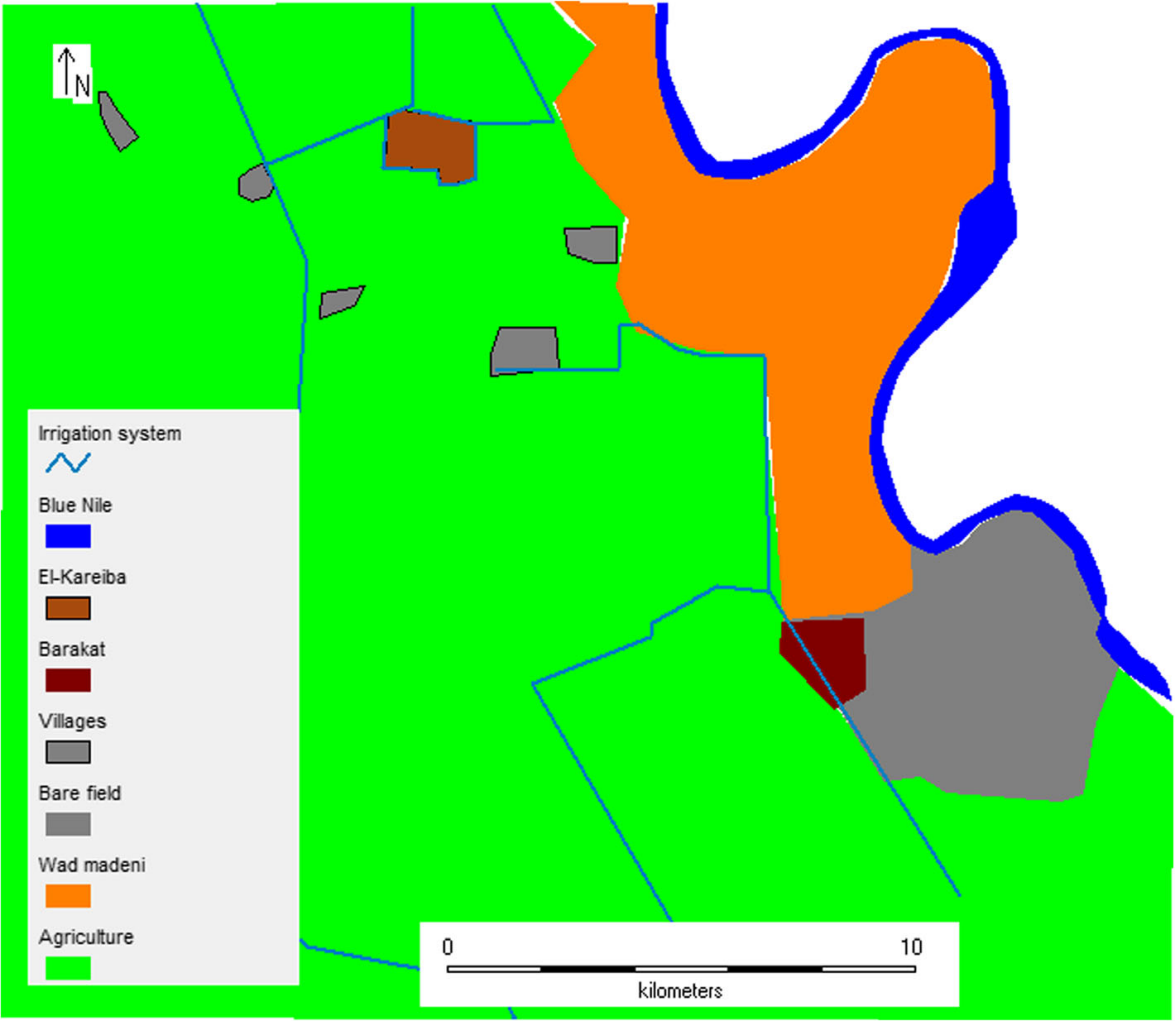
A map showing the study sites

This study was carried out between February and September 2011 in the Barakat (33.32 °N; 14.18 °E) and El-Kareiba (33.27 °N; 14.24 °E) villages, located approximately 20 km from the town of Wad Madani, central Sudan (Fig. 1). The area has a dry savanna climate with an estimated annual rainfall of 140–225 mm. Relative humidity fluctuates between 37 and 86%. There are three annual seasons: a short rainy season from July to September, a dry cool season from November to March and a hot dry season from April to June. The mean temperature varies between 15 °C and 21 °C in the cool dry season and 32 °C to 42 °C in the hot dry season (Sudan Metrological Service, unpublished data).

### Larval surveys

A portable geolocation system (Magellan Triton 400, PJM technology Industry Co., Ltd, Shenzhen Pengjin Technology Co., Ltd, Shenzhen China) was used to mark the breeding habitats for each survey. Surveys were conducted bi-monthly and the same habitats were visited each time.

The breeding sites were categorised as follows: (1) irrigation channels (canals used to direct water in paddy plots from the main channel); (2) broken pipes (water leakage from pipes used for drinking water); (3) animal hoof prints (which provide waterlogged sections of land resulting in small mosquito breeding sites); (4) barrels (large containers used for storing water for domestic purposes, pool storage, Jars); and (5) open basins (used for storing water for construction purposes). Hoof prints, tyre tracks and broken water pipes were considered as temporary habitats, while barrels, open basins and irrigation canals were considered as permanent productive habitats. Land use types were described as: (1) farmland (land used for the production of food crops); (2) pastures (land covered with low plants suitable and used for grazing animals); and (3) roads (land used as a passage for animals, people and vehicles).

A standard dipper (350 mls) (BioQuip Products, Inc. California, USA) was used for estimating the number of mosquito larvae, pupae and aquatic invertebrates in each habitat. A total of 20 dips were made in each habitat every visit. The immature stages, 1^st^ and 2^nd^ instars, were grouped together as earlier instars, while the 3^rd^ and 4^th^ instars were grouped together as late instars. All *Anopheles* larvae and pupae were collected in 25 ml vials with 75% alcohol and transported to insectaries at the Blue Nile National Institute for Communicable Diseases (BNNICD), University of Gezira, Wad Madani, Sudan. The marked coordinates were input into ArcView version 10.4 (ESRI, 380 New York Street, Redlands, CA 92373–8100 USA) for analysis and for drawing maps.

### Habitat characterisation

In each survey, the habitat type, habitat size, vegetation cover (percentage/proportion of the vegetation covering the larval habitat surface), presence of predators and larval density by instars were recorded.

### Data analysis

The data obtained from the entomological surveys were analysed using JMP version 5.0.1.2 (SAS Institute, Inc., NC, USA). The means of larval densities were compared between the different seasons and sites using the Tukey-Kramer test with analysis of variance (ANOVA). Associations between habitat characteristics (Grass cover, polluted habitats, turbidity, shading, Algae and predator) and larval densities were tested using logistic regression analysis. Analysis was not performed by instars because the variations between and within instars were very large and hence could distort the biological meaning of obtained results and its interpretation.

## Results

### Species composition and productivity

A total of 5 525 larvae were sampled from 331 breeding habitats and sent to the insectary to be identified to species level using a morphological key developed by Gillies and Coetzee [19]. Of the sampled larvae, 2 617 (47.37%) were of the *Culex*species, 2 600 (47.06%) were *Anopheles* and 308 (5.57%) were *Aedes*. Immature culicine mosquitoes were taxonomically identified as *Culex antennatus* (42%), *Cx. Quinquefasciatus* (25%), *Cx. simpsoni* (14%), *Cx. Tritaeniorhynchus* (8%), *Cx. Theileri* (5%), *Cx. musarum* (4%) and *Cx. pipiens* (2%). Anopheline mosquitoes were taxonomically identified as *An. arabiensis* (38.0%), *An. funestus* (27.0%), *An. Rufipes* (24.5%), *An. Pharoensis* (9.5%)*, An. Nili* (0.5%) and *An. dattali* (0.5%). A total of 308 *Aedes* mosquitoes were identified as *Aedes aegypti* (*n* = 47, 15.3% in Barakat; *n* = 13, 4.2% in El-Kareiba) from positive breeding sites, which were reported during the cool dry season. In this study, adult habitat productivity (number of adult females emerged/m^2^) was not homogeneous. The highest productivity was observed in irrigation channels (0.78 females/m^2^) for *Anopheles*, and in septic tanks (2.86 females/m^2^) for *Culex* and (0.86 females/m^2^) for *Aedes* (Tables 1 and 2).

**Table 1:** Habitat productivity (emerged adult females/m^2^) for different mosquito genera in different habitat types in Gezira, central Sudan, February – September 2011 (Mean ± SE of mosquitoes)

Habitat type	No. of habitats	*Anopheles* spp.	*Culex* spp.	*Aedes* spp.
Jars	16	0.0	0.0	0.56 ± 0.5
Septic tanks	7	0.0	2.86 ± 2.86	0.86 ± 0.9
Barrels	10	0.6 ± 0.43	0.1 ± 0.1	0.0
Big open water storage	11	0.18 ± 0.18	0.0	0.0
Irrigation channels	46	0.78 ± 0.29	0.5 ± 0.25	0.0
Animal hoof prints	47	0.15 ± 0.1	0.81 ± 0.53	0.0
Leakage of pipe	142	0.42 ± 0.12	1.079 ± 0.52	0.014 ± 0.01
Tyre tracks	43	0.093 ± 0.06	0.0	0.0

**Table 2:** Presence of different mosquito larvae in the different habitat types in Barakat and El-Kareiba, Gezira, Central Sudan, February – September, 2011

		Barakat			El-Kareiba		
Habitat type	Habitats (no.)	*Anopheles* spp. (%)	*Culex* spp. (%)	*Aedes* spp. (%)	*Anopheles* spp*.* (%)	*Culex* spp. (%)	*Aedes* spp. (%)
Leakage of pipe	142	661 (40.0)	1 240 (56.5)	58 (33.9)	577 (60.8)	187 (44.3)	24 (17.5)
Animal hoof prints	47	223 (13.5)	138 (6.3)	4 (2.3)	194 (20.4)	74 (17.5)	3 (2.2%)
Irrigation channels	46	610 (37.0)	512 (23.3)	46 (26.9)	NA	NA	NA
Tyre tracks	43	88 (5.3)	75 (3.4)	30 (17.5)	130 (13.7)	0.0	0.0
Jars	16	0.0	5 (0.2)	33 (19.3)	NA	NA	NA
Big open water storage	11	41 (2.5)	28 (1.3)	0.0	28 (3.0)	2 (0.5)	0.0
Barrels	10	28 (1.7)	26 (1.2)	0.0	20 (2.1)	59 (14.0)	0.0
Septic tanks	7	0	171 (7.8)	0.0	0.0	100 (23.7)	110 (80.3)
*P*		0.0003	0.0300	0.6000	0.2000	0.0080	0.2000

*NA* Not applicable; habitat type not found

### Habitat characteristics

A total of 331 breeding sites were visited. The most common type of larval habitat was leaking water pipes. The presence of *Anopheles* larvae was significantly associated with open habitats (exposure to sunlight) (*χ* ^2^ = 5.237, *P* = 0.0221), turbidity (*χ* ^2^ = 2.45, *P* = 0.1176), presence of pollution (*χ* ^2^ = 0.35, *P* = 0.5522), grass cover (*χ* ^2^ = 9.12, *P* = 0.0025), presence of algae (*χ* ^2^ = 11.897, *P* = 0.0026) and abundance of predators (*χ* ^2^ = 29.92, *P* < 0.0001) (Table 3).

**Table 3:** Associations between presence ofmosquito larvae and habitat characteristics determined using logistic regression analysis

Mosquito species	Characteristic	Chi-square	*P*
Anopheles larvae	Grass cover	9.12	0.0025
	Non-polluted habitats	0.35	0.5522
	Turbidity	2.45	0.1176
	Habitat shading	0.30	0.5868
	Algae	11.897	0.0026
	Predators	29.92	<0.0001
Culex larvae	Predators	25.86	0.0001
	Algae	9.34	0.0022
Aedes larvae	Grass cover	9.12	0.0025
	Open habitat	9.34	0.0129

The logistic regression analysis showed that the presence of *Anopheles* larvae was found to decrease with the abundance of predators (backswimmers, tadpoles) and grass cover. The increase of Culicinaelarvae was associated with habitats characterised by low or absence of turbidity (*χ* ^2^ = 9.34; *P* = 0.0022) and absence of predators (*χ* ^2^ = 25.86; *P* < 0.0001). The presence of *Aedes* larvae was associated with open habitats (*χ* ^2^ = 934; *P* = 0.0129) and no grass cover (*χ* ^2^ = 9.12; *P* = 0.0025) (Table 3).

### Determination of positive and negative mosquito breeding sites

**Fig. 2:**
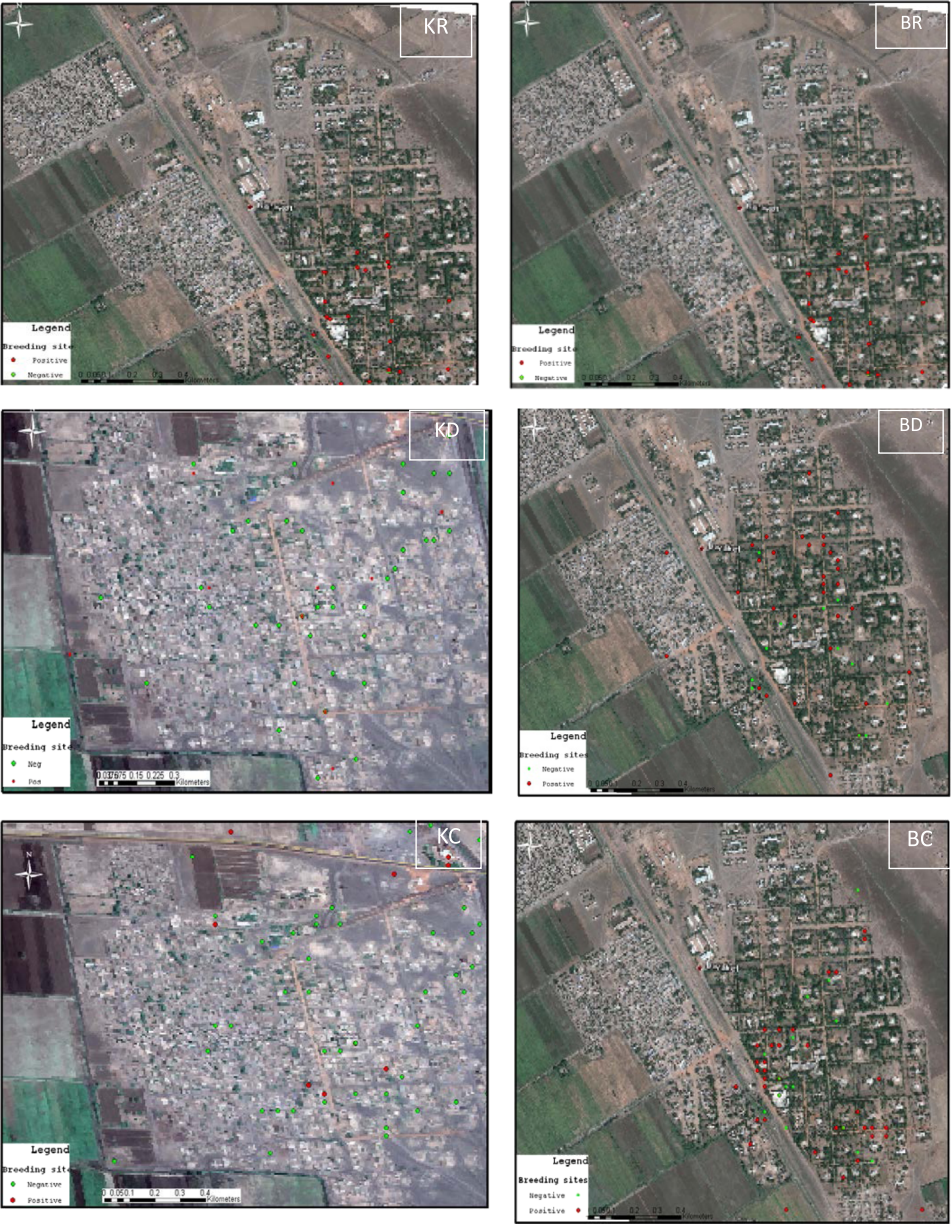
Distribution of breeding sites during the three seasons. KC: El-Kareiba cold season, KD: El-Kareiba dry season; KR: El-Kareiba rainy season; BC: Barakat cold season; BD: Barakat dry season; BR: Barakat rainy season

Eight habitat types were inspected in the two study sites. Among them, 51.6% (*n* = 166) were found to be positive mosquito breeding sites. A significant difference was observed over the seasons between the positive and negative breeding sites in both Barakat (*χ* ^2^ = 10.641, *P* = 0.0050) and El-Kareiba (*χ* ^2^ = 23.754, *P* < 0.0001) (Fig. 2). The highest number of positive mosquito breeding sites was recorded during the rainy season in both Barakat (96.4%; 27/28) and El-Kareiba (65.6%; 21/32). A significant difference was observed in *Anopheles* larvae presence in the different habitats in Barakat. The most preferred habitat of *Anopheles* larvae was leaking water pipes (40%) (Table 2).

### Mosquito larval abundance and spatial-temporal distribution among the different habitat types

**Fig. 3:**
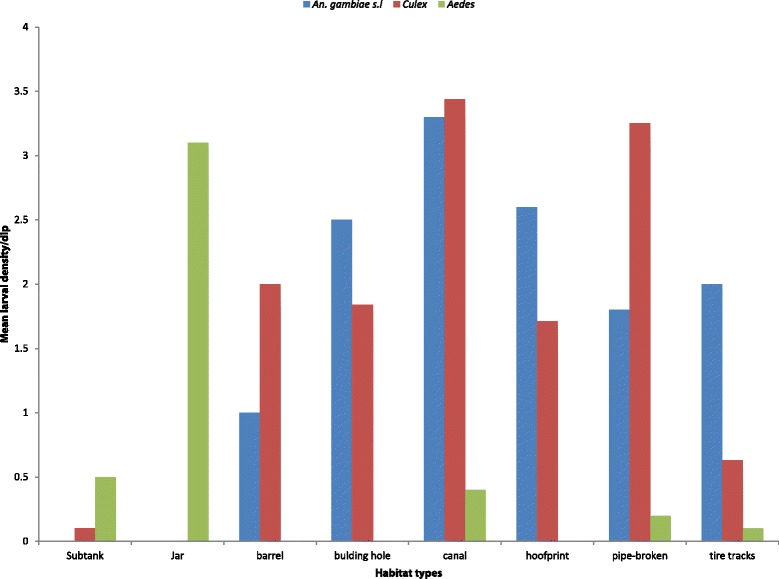
Mean larval densities of *Anopheles*, *Culex* and *Aedes* mosquitoes by habitat type

**Fig. 4:**
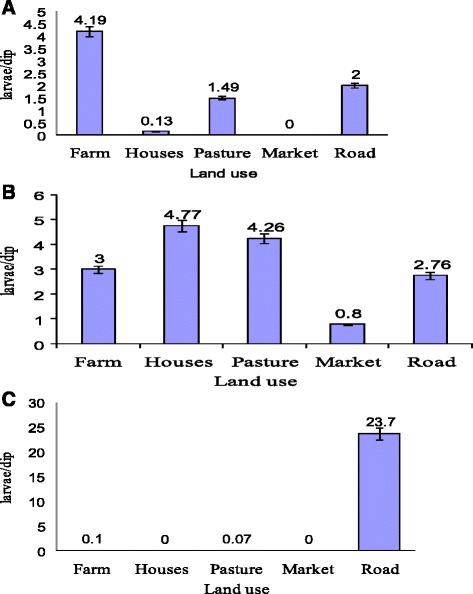
Densities of *Anopheles* (**a**), *Culex* (**b**) and *Aedes* (**c**) mosquitoes in the different types of land in Barakat and El-Kareiba

Overall, 2 617 *Culex* larvae were collected from eight larval habitat types: leaking water pipes (54.53%), irrigation channels (19.55%), animal hoof prints (8.10%), septic tanks (10.36%), tyre tracks (4.02%), jars (0.19%) and water tanks (3.25%). *Anopheles* larvae were collected from six habitat types: leaking water pipes (47.61%), irrigation channels (23.48%), animal hoof prints (16.03%), tyre tracks (8.38%), Water pools (2.42%) and water tanks (1.85%) (Fig. 3 and Table 1). *Ae. Aegypti* are reported though in low density dominated in Jars and Septic tanks (Fig. 3 and Table 1). About 78.1% of all positive breeding sites were located on farms, followed by roads (12.3%) and houses (9.6%). Farmland type was significantly associated with positive mosquito breeding sites (*χ* ^2^ = 15.902, *P* = 0.0032) (Fig. 4). The density of *Anopheles* larvae (larvae per dip) on farmland was 4.2 compared to 2.0 on roads and 1.4 on pastures. *Aedes* mosquitoes preferred domestic land surroundings due to the high abundance of containers and tanks found on this area, which are all suitable breeding sites (Fig. 4).

### Effectofseasonsonsites

During this study, the proportion of positive mosquito breedingsiteswassignificantly higherduringtherainyseason than in dry season for both Barakat (*F* =16, df = 2, *P* = 0.0049) and El- Kareiba (*F* = 16, df = 2, *P* < 0.0001). The proportions of positive breeding sites during the rainy season were 96.4% and 65.6% in Barakat and El-Kareiba, respectively. The proportion of positive breeding sites during the cool dry season in Barakat (4.7) was almost four or three times compared to El-Kareiba (1.7) for *Culex* spp.. For *Anopheles* spp. it was 2.69 in Barakat and 1.49 in El-Kareiba while for *Aedes* spp*.* it was 0.30 in Barakat and 0.35 in El-Kareiba for Cool dry season (Table 6).

The densities of *Culex*, *Anopheles* and *Aedes* larvae during the different seasons in the two sites are shown in Tables 5 and 6. There was a significant difference in the larval densities of *Anopheles* mosquitoes in the different seasons and at different sites. However, no significant difference was observed in the density of both *Culex* and *Aedes* larvae over the seasons, except the density of *Aedes* larvae in El-Kareiba, where it was found to be restricted to the cool dry season. The highest larval density was observed during the rainy season for both *Anopheles* and *Culex* species, while the peak of *Aedes* larval density occurred during the cool dry season in El-Kareiba (Tables 4 and 5). The larval densities of both the *Culex* and *Anopheles* species were significantly higher in the Barakat irrigated area compared to the El-Kareiba site (Table 6).

**Table 4:** Numbers of mosquito larval habitats and their characteristics in Barakat and El-Kareiba, Gezira, Sudan, February – September, 2011

Characteristics	Barakat			El-Kareiba		
	*Anopheles* spp. (%)	*Culex* spp*.* (%)	*Aedes* spp*.* (%)	*Anopheles* spp. (%)	*Culex* spp*.* (%)	*Aedes* spp*.* (%)
Habitat location						
Indoor	337 (20.4)	244 (11.1)	0.0	0.0	0.0	0.0
Outdoor	1 314 (79.6)	1 951 (88.9)	171 (100)	949 (100)	1 328 (100)	43 (100)
Presence of larvae						
Negative	0.0	0.0	0.0	0.0	0.0	0.0
Positive	1 651 (100)	2 195 (100)	171 (100)	949 (100)	1 328 (100)	43 (100)
Shading						
No	1 098 (66.1)	1 807 (82.4)	111 (65.0)	949 (100)	328 (24.7)	27 (62.8)
Yes	553 (33.5)	388 (17.7)	60 (35.1)	0.0	1 000 (75.3)	16 (37.2)
Water turbidity						
Non-turbid	1 048 (63.5)	1 567 (71.4)	129 (75.5)	624 (65.8)	1 251 (94.2)	
Low turbidity	529 (32.0)	623 (28.4)	20 (11.7)	276 (29.1)	77 (5.8)	27 (62.8)
High turbidity	74 (4.5)	5 (0.2)	22 (12.9)	49 (5.0)	0.0	16 (37.0)
Grass presence						
Yes	1 283 (77.7)	1 592 (72.5)	76 (44.5)	444 (46.8)	423 (31.9)	3 (6.9)
No	368 (22.3)	603 (27.5)	95 (55.6%)	405 (42.7)	1 085 (81.7)	40 (93.0)
Algae presence						
Yes	1 217 (73.7)	1 555 (70.9)	119 (69.7)	661 (69.65)	249 (18.8)	3 (5.0)
No	434 (26.3)	640 (29.2)	52 (30.5)	288 (30.4)	1 079 (81.3)	40 (93.0)
Larval control						
Yes	16 (1.0)	24 (1.1)	3 (1.8)	0.0	0.0	0.0
No	1 635 (99.0)	2 171 (98.9)	168 (98.3)	949 (100)	1 328 (100)	43 (100)

**Table 5:** Mean larval densities of *Culex*, *Anopheles* and *Aedes* mosquitoes in the different seasons in Barakat and El-Kareiba, Gezira, Sudan, February – September, 2011

	Barakat				El-Kareiba			
		Mosquito species				Mosquito species		
Season	No. of habitats	*Culex* spp*.* ± SE	*Anopheles* spp*. ±* SE	*Aedes* spp*. ±*SE	No. of habitats	*Culex* spp*. ±* SE	*Anopheles* spp*. ±* SE	*Aedes* spp. *±* SE
Cool dry	66	4.4 (1.89)	3.0 (0.52)	0.2 (0.17)	75	2.8 (1.73)	0.4 (0.39)	4.1 (29.32)
Hot dry	56	2.8 (2.05)	1.7 (0.56)	0.5 (0.18)	65	0.3 (1.86)	1.1 (0.42)	0.0 (31.50)
Rainy	28	9.2 (2.90)	4.0 (0.79)	0.0	41	2.2 (2.34)	4.3 (0.53)	0.063 (69)
*P*		0.1951	0.0497	0.3093		0.1598	<0.0001	0.0250

**Table 6:** Density (larvae/dip) of *Culex, Anopheles* and *Aedes* mosquitoes in the two sites in Gezira, Sudan, February – September 2011

Site	No. of habitats	*Culex* ± SE	*Anopheles* ± SE	*Aedes* ± SE
Barakat	150	4.7 ± 1.25	2.69 ± 0.35	0.3 ± 0.12
El-Kareiba	172	1.7 ± 1.1	1.46 ± 0.29	0.35 ± 0.32
*P*		0.0516	0.0068	0.9000

## Discussion

The findings of this study demonstrate that the main potential mosquito breeding habitats in urban areas are leaking water pipes and irrigation channels in agricultural schemes during the dry season. Of all suitable breeding sites visited, less than 52% were found to be positive mosquito breeding sites, and this percentage was found to decrease during the dry season. In Africa, the larval source management have shown to be very effective in all small scale and urban areas implemented [20] In western Kenya highlands and urban area of Dar-es-salaam, Tanzania the larval source management have shown a great impact when managed alone or in combination with IRS and LLINs implementation [19]. Decline of adult vectors and transmission of malaria parasites was found vivid [19]. Its a proof that, if LSM is implemented in large scale and integrated with LLINs and IRS, a great improvement shall be seen in malaria control efforts than we are getting for IRS and LLINs alone.

The species composition in this study was: 47.06% were *Anopheles* species, 47.37% were *Culex* species and the remaining 5.57% were *Aedes* species, which were identified as *Ae. aegypti.* These findings are similar to what was reported by previous studies on *Ae. aegypti* abundance, only differing in seasonality and habitat types [2, 6, 21].

The productivity of the three mosquito genera was found to be 0.78 females/m^2^, 2.86 females/m^2^ and 0.86 females/m^2^ for *Anopheles spp., Culex spp* and *Aedes* spp., respectively. This is the very first time that productivity of each genus has been reported in this particular location. Productivity was found to be habitat-species specific. High productivity of *Anopheles* spp. was found in irrigation channels, and high productivity of *Culex spp*and *Aedes* spp. was found in septic tanks. Productivity was also recorded for each genus in other habitat types, but at lower levels.

During the rainy season, the average proportion of positive mosquito breeding sites was found to be over 81% in both study areas. This results in differences in mosquito densities between dry and rainy seasons, which could be attributed to the availability of water and fluctuations in temperature and relative humidity [3, 6, 14, 15, 22, 23]. In Sudan, adult mosquito density, i.e., *An. arabiensis*, has been shown to either decrease or completely disappear during the hot dry season [10, 24]. *An. arabiensis* was is the dominant *Anopheles* species in Sudan [10, 12, 13] and was is considered to be the main malaria vector in the country [10, 12, 25, 26].

Importantly, this study documented for the first time the presence of *An. funestus* in Wad Madani. The favourable habitat for this species was found to be vegetated irrigation channels. In equatorial areas, the peak of *An. funestus adult population* is at the end of the rainy and beginning of the dry season [27]. Seven *Culex* species were recorded in this study, which was contrary to fewer species reported by previous reports [9]. All *Culex* species found in this study were reported by Lewis reported all Culex species found in this study in 1956 [13]. *Ae. aegypti* was the only species of the *Aedes* genus reported in the study areas during the study period. It was sampled permanently in septic tanks and jars. It is known that the local habitat profile of *Ae. aegypti* is associated with human dwellings and community socio-economic factors such as well-being and poverty [28, 29].

In the present study, immature mosquitoes found in turbid water were almost always of the *Culex* species, which is similar to the findings of Devi and Jauhari [30]. Anopheline mosquitoes preferred to breed in clear water, which was similar to the findings of Robert et al. [31]. Significant differences were observed among the three seasons for *Anopheles* larvae. Similar findings have shown that seasonality has an effect on *An. gambiae sensu lato* dynamics and abundance [15, 16], and could demonstrate that anopheline mosquitoes are more sensitive to climatic variables, mainly temperature and relative humidity [21, 27, 32]. In this study, peak vector densities were observed during the rainy season and this result agrees with other observations from the same and other dry savanna areas of eastern and central Sudan [10, 12, 33]. It is interesting to note that the most productive breeding site for anopheline mosquitoes found over the different seasons was irrigation channels, and this may explain why the density of *Anopheles* larvae was significantly higher in irrigation area of Barakat as compared to El-Kareiba.

The findings of this study show that that, targeting mosquito species of different genera can be done by LSM implementation in their specific habitats. This would make control efforts for each genus easier and more economical. Similar findings were found in previous studies conducted in western Kenyan highlands [15, 16] and Dar es Salaam, Tanzania [20] thought the coverage of programmes were limited to small scale area coverage.

## Conclusions

Frequent updating of the mosquito species composition and dynamics in the Gezira Scheme are considered necessary for effect control plan. A low mosquito larval density was observed during the dry season in this study, which suggests that it would be cost-effective to conduct larval control in the dry season. Currently, vector interventions can be integrated with adult mosquito control (use of long-lasting insecticidal nets and indoor residual spraying) during the dry season. The main breeding sites were identified for each genus and earmarked for the best LSM in this area which suggested dry season better time for timed LSM.

## Additional file

Additional file 1: Multilingual abstracts in the five official working languages of the United Nations. (PDF 768 kb)

## Abbreviations

BNNICD: Blue Nile National Institute for Communicable Diseases
Df: Degree of freedom
F: F-statistics for ANOVA output
IRS: Indoor residual spray
LLINs: Long lasting Insecticidal nets
LMS: Larval source management
