# Supplementary material for: Characterisation of larval habitats, species composition and factors associated with the seasonal abundance of mosquito fauna in Gezira, Sudan
Source: Infect Dis Poverty. 2017 Feb 8;6:23. doi: 10.1186/s40249-017-0242-1 (PMC5297020; doi:10.1186/s40249-017-0242-1)
Supplement: Additional file 1: — Multilingual abstracts in the five official working languages of the United Nations. (PDF 768 kb) [file 40249_2017_242_MOESM1_ESM.pdf]

Translation of the abstract into the five official working languages of the United Nations

خصائص مواطن اليرقات والتركيب النوعي والعوامل المرتبطة بالوفرة الموسمية لحيوانات البعوض في الجزيرة بدولة السودان

مصطفى م. محجوب، الينينجايا ج. كويكا، يوسف إ. حمدان

**الملخص**

**الخلفية:** تُعد إدارة مصادر اليرقات، والتي تتطلب فهمًا لبيئة وتكوين حيوانات البعوض المحلية، عاملاً مهمًا في نجاح برامج مكافحة ناقلات الأمراض. وقد أجريت هذه الدراسة لفهم توزيع مواطن اليرقات والتكوين النوعي والعوامل المرتبطة بالوفرة الموسمية ليرقات البعوض في نظام ري الجزيرة في ولاية الجزيرة بوسط السودان.

**الأساليب:** تم إجراء علميات مسح متقاطعة في مجتمعات بركات (مجتمعات حضرية) والكريبه (مجتمعات شبه حضرية) في واد مدني بولاية الجزيرة. وقد تم استخدام غطاس معياري لأخذ عينات من اليرقات في جميع المواقع المحتملة لتكاثرها وتم استخدام أوعية من المينا لفرز العينات. وتم تمييز المواطن باستخدام خصائص مادية وتم تحديد جميع عينات اليرقات شكليًا.

**النتائج:** تم مسح إجمالي عدد 331 موطنًا من مواطن اليرقات، تبين أن من بينها 166 موطنًا تمثل مواقع تكاثر إيجابية لأنواع البعوضات الأنوفيلة (56.78%) والبعوضاوات (29.67%) والزاعجة (13.55%). وتم جمع إجمالي عدد 5525 يرقة تم تصنيفها من نوع بعوضة (2617، 47.37%) وأنوفيلة (2600، 47.06%) وزاعجة (308، 5.57%). كان هناك عددًا كبيرًا من المواطن الإيجابية خلال موسم الأمطار بينما أفادت التقارير عن أن أدنى نسبة كانت خلال الموسم الحار الجاف، في كلا موقعي الدراسة (بركات $\chi^2$=10.641، $P$=0.009)، الكريبه [$\chi^2$=23.765، $P$=0.0001]). وكان الموقع الرئيسي لتكاثر يرقات بعوضات الأنوفيلة أنابيب المياه المتسربة (51.5%)، تليها قنوات الري (34.2%) وآثار حوافر الحيوانات (6.4%) وآثار الإطارات (5.5%) وخزانات المياه (2.4%). وأظهر تحليل الانحدار اللوجستي أن وفرة يرقات بعوضة الأنوفيلة قد انخفضت بسبب وجود الحيوانات المفترسة (البق السابح ظهريًا والضفادع الصغيرة) والغطاء العشبي. ولم تكن إنتاجية البالغين (عدد الإناث البالغات الظاهر لكل متر مربع) متجانسة لجميع المواطن، حيث تبين أن أعلى إنتاجية كانت في قنوات الري (0.78 أنثى / م2) لبعوضة الأنوفيلة والبلاعات (2.86 أنثى / م2) لفصيلة البعوضات و(0.86 أنثى / 62) لبعوضة الزاعجة. كما تبين أن ارابينسيس الأنوفيلة هي فيصلة الأنوفيلة المهيمنة. وقد وثقت هذه الدراسة وجود أنوفيلة مشؤومة في وسط السودان لأول مرة.

**الاستنتاجات:** يُنصح بصيانة أنابيب المياه المتسربة والري المتقطع لإدارة مصادر اليرقات، حيث تمثل هذه المواطن التي تم مسحها المصدر الرئيسي للحفاظ على أسراب البعوض المحلي خلال الموسم الحار الجاف.

Translated from English version into Arabic by Dr. Manale Elewah, through

TRANSLATORS
WITHOUT BORDERS

**苏丹杰济拉地区蚊类幼虫孳生地特征描述、物种构成和季节消长规律的相关因素**

Mostafa M. Mahgoub, Eliningaya J. Kweka, Yousif. E. Himeidan

**摘要**

**引言：**幼虫源头管理(LSM)是媒介控制策略中的一项重要措施，它需要充分了解当地蚊媒的组成和生态习性。本研究是对苏丹中部杰济拉州杰济拉灌溉项目中的蚊类幼虫孳生地的分布、蚊种构成，以及季节消长规律相关因素进行了调查。

**方法：**本研究在杰济拉州瓦德迈达尼的巴拉卡特（城镇）和El-Kareiba（半城镇）进行横断面幼虫调查。在所有可能的孳生地用标准长柄勺进行幼虫进行取样，并用搪瓷碗对幼虫进行

分类。根据物理特征对孳生地进行特征描述，利用形态特征对所有幼虫标本进行鉴定。
**结果：**共计调查331个幼虫孳生地，其中在166个孳生地发现幼虫，蚊种所占比例分别为按蚊属56.78%, 库蚊29.67%和伊蚊属13.55%。共收集5 525条幼虫，分别隶属于库蚊属（2 617条，47.37%），按蚊属(2 600条，47.06%)和伊蚊属（308条，5.57%)。所有的调查点在雨季有大量的阳性孳生地，而在热的旱季最少(Barakat [$\chi^2$=10.641, *P*=0.009], El-Kareiba [$\chi^2$=23.765, *P*=0.0001])。按蚊幼虫的主要孳生地是漏水的水管(51.5%)、灌溉渠(34.2%)、蹄印(6.4%)、轮胎辙痕(5.5%)和水箱(2.4%)。Logistic回归分析表明，按蚊幼虫密度随着捕食者（仰泳蝽、蝌蚪）和草地覆盖的出现而降低。所有孳生地成蚊生殖力（雌蚊数量/ $m^2$）是不同的，灌溉渠的按蚊(0.78雌蚊/$m^2$)、化粪池的库蚊(2.86 雌蚊/$m^2$)和伊蚊(0.86 雌蚊/$m^2$)生殖力最高。本研究发现阿拉伯按蚊（*Anopheles arabiensis*）是优势蚊种，并且首次记录了在苏丹中部有催命按蚊（*An. funestus*）。
结论：本研究建议在幼虫源头管理时对漏水的水管进行维修和采用间歇灌溉的办法，因为调查所发现的孳生地是在干旱季节维持当地蚊类种群的主要来源。

Translated from English version into Chinese by Xin-Yu Feng, edited by Pin Yang, through

CHINESE CENTER FOR DISEASE CONTROL AND PREVENTION
NATIONAL INSTITUTE OF PARASITIC DISEASES

## Caractérisation des habitats larvaires, composition spécifique et facteurs associés à l’abondance saisonnière des moustiques dans la Gezira, au Soudan

Mostafa M. Mahgoub, Eliningaya J. Kweka, Yousif E. Himeidan

**Résumé**
**Contexte :** La gestion des gîtes larvaires (GGL [OMS]) est un paramètre important pour la réussite des programmes de lutte contre les vecteurs, qui nécessite la compréhension de l’écologie et de la répartition locale des espèces de moustiques. La présente étude a été menée dans le but de comprendre la distribution des habitats larvaires, la composition spécifique et les facteurs associés à l’abondance saisonnière de moustiques dans le Programme d’irrigation de la Gezira, dans l’état de Gezira (Al-Jazirah) dans le centre du Soudan.
**Méthodes:** Des recensements transversaux de larves ont été effectués dans les quartiers de Barakat (milieu urbain) et El-Kareiba (milieu semi-urbain) à Wad Madani dans l’état de Gezira. Un godet de prélèvement standard a été utilisé pour échantillonner les larves dans tous les sites de reproduction possibles et des bols en émail pour les trier. Les habitats ont été caractérisés en utilisant des paramètres physiques et tous les spécimens de larves ont fait l’objet d’une détermination morphologique.
**Résultats :** Au total, 331 habitats larvaires ont été inspectés et 166 se sont avérés des sites de reproduction d’*Anopheles* (56,78 %), Culicinae (29,67 %) et *Aedes* (13,55 %). Les 5525 larves collectées ont été identifiées comme des *Culex* (2617, 47,37 %), *Anopheles* (2600, 47,06 %) et *Aedes* (308, 5,57 %). De nombreux habitats positifs ont été découverts pendant la saison des pluies, tandis que la proportion la plus faible a été observée pendant la saison sèche sur les deux sites d’études (Barakat [$\chi^2$ = 10,641, *P* = 0,009], El-Kareiba [$\chi^2$ = 23,765, *P* = 0,0001]). Les conduites d’eau percées représentent le principal site de reproduction pour les larves d’*Anopheles* (51,5 %),

suivies des canaux d’irrigation (34,2 %), des empreintes de sabots (6,4 %) et de pneus (5,5 %) et des citernes d’eau (2,4 %). Une analyse de régression logistique a montré que l’abondance de larves d’*Anopheles* était réduite par la présence de prédateurs (notonectes, têtards) et la couverture végétale. La productivité des adultes (nombre de femelles adultes écloses/$m^2$) n’était pas homogène entre les habitats : la plus forte productivité a été trouvée dans les canaux d’irrigation (0,78 femelle/$m^2$) pour *Anopheles* et dans les fosses septiques pour les Culicinae (2,86 femelles/$m^2$) et *Aedes* (0,86 femelle/$m^2$). *Anopheles arabiensis* est l’espèce d’anophèle dominante. Cette étude a enregistré pour la première fois la présence d’*An. funestus* au Soudan central.
**Conclusions :** La réparation des conduites d’eau percées et l’adoption de l’irrigation intermittente sont deux mesures recommandées pour la gestion des gîtes larvaires. C’est en effet principalement dans ces habitats que les populations locales de moustiques peuvent se maintenir pendant la saison sèche.

Translated from English version into French by Suzanne Assenat, through

TRANSLATORS
WITHOUT BORDERS

**Характеристика среды обитания личинок, видовой состав и факторы, связанные с сезонным обилием комаров фауны в Гезире, Судан**

Mostafa M. Mahgoub, Eliningaya J. Kweka, Yousif. E. Himeidan

**Реферат**
**Фон:** Управление личиночной источник (ЛСМ), которая требует понимания экологии и состав местных комаров фауны, является важным параметром успешной реализации программ. Настоящее исследование проводилось с целью понять распределение среды обитания личинок, видовой состав и факторы, связанные с сезонным обилием личинок комаров в схема Гезира орошения в штате Эль-Гезира, Центральном Судане.
**Методы:** Кросс-секционных обследований личинок проводились в сообществах Бараката (городских) и El-Kareiba (поселках городского типа), вгороде Вад Мадани Гезира. Стандартный ковш используется для отбора проб личинок во всех возможных местах гнездования и эмалированные миски были использованы для сортировки личинок. Местообитания характеризуются использованием физических особенностей и образецы всех личинок были идентифицированы морфологически.
**Результаты:** В общей сложности 331 среды обитания личинок опрошенных, из которых 166 были найдены, чтобы быть положительным размножения *Anopheles* (56.78%), *Culicinae* (29.67%) и *Aedes* (13.55%) видов. В общей сложности собрал 5 525 личинки были классифицированы как *Culex* (2 617, 47.37%), *Anopheles* (2 600, 47.06%) и *Aedes* (308, 5.57%). Там было большое количество положительных обитания во время сезона дождей, в то время как наименьшая доля сообщалось, во время жаркого сухого сезона, в обоих исследуемых участках (Баракат [$\chi^2$=10.641, $P$=0,009], El-Kareiba [$\chi^2$=23.765, $P$=0,0001]).

Основным местом для размножения личинок комаров *Anopheles* была утечка воды трубы (51.5%), далее за ним следуют оросительные каналы (34.2%), отпечатки копыт (6.4%), следы шин (5,5%) и цистерны с водой (2.4%). Анализ логистической регрессии показал, что численность личинок Малярийных была снижена на присутствие хищников (гладыши, головастики) и травяной покров. Взрослые производительность (количество взрослых самок появились/м2) не был однородным для всех местообитаний; самая высокая производительность труда была найдена в оросительных каналах (0.78 женщин /м2) для комаров *Anopheles*, и в септиках (2.86 самок /м2) для *Culicinae* и (0.86 женщин/м2) для комаров *Aedes.* Комаров *Anopheles Arabiensis* из оказался доминирующих видов *Anopheles*. Это исследование показало *An. funestus* в Центральном Судане впервые.
**Заключение:** Поддержание протечки водопроводных труб и принятия прерывистого орошения рекомендованы для ЛСМ, как этих обследованных местообитаний являются основным источником сохранения местной популяции комаров во время жаркого сухого сезона.

Translated from English version into Russian by Hao-Qi Zhang,

**Caracterización de los hábitats de larvas, composición de especies y factores asociados con la abundancia estacional de la fauna de mosquitos en Gezira, Sudán**

Mostafa M. Mahgoub, Eliningaya J. Kweka, Yousif. E. Himeidan

**Resumen**

**Antecedentes:** La gestión de las fuentes de larvas (LSM), que requiere de un entendimiento de la ecología y composición de la fauna local de mosquitos, es un parámetro importante para el éxito de los programas de control de vectores. El presente estudio se llevó a cabo para comprender la distribución de los hábitats de larvas, la composición de especies y los factores asociados con la abundancia estacional de larvas de mosquito en el esquema de irrigación de Gezira en el estado de Gezira en la zona central de Sudán.
**Métodos:** Se llevaron a cabo encuestas transversales en las comunidades de Barakat (urbana) y El-Kareiba (semi-urbana), en Wad Madani, Gezira. Se utilizó un cazo de muestreo estándar para las muestras de larvas en todos los posibles sitios de reproducción y se utilizaron cuencos esmaltados para la clasificación de las larvas. Se caracterizó a los hábitats mediante el uso de características físicas y se identificó morfológicamente a todos los especímenes de larvas.
**Resultados:** Se encuestó un total de 331 hábitats de larvas, de los cuales 166 fueron sitios de reproducción positivos para las especies *Anopheles* (56,78%), Culicinae (29,67%) y *Aedes* (13,55%). Se caracterizaron un total de 5525 larvas recolectadas como *Culex* (2617, 47,37%), *Anopheles* (2600, 47,06%) y *Aedes* (308, 5,57%). Hubo una gran cantidad de hábitats positivos durante la temporada de lluvias, mientras que la proporción más baja se reportó durante la temporada seca de calor, en ambos sitios del estudio (Barakat [$\chi^2$=10,641, $P$=0,009], El-Kareiba [$\chi^2$=23,765, $P$=0,0001]). El principal sitio de reproducción para las larvas *Anopheles* fueron tuberías con fugas (51,5%), seguidos de canales de irrigación (34,2%), huellas de animales (6,4%), huellas de vehículos (5,5%) y tanques de agua (2,4%). Un análisis de regresión logística mostró que la abundancia de las larvas

*Anopheles* se veía reducida por la presencia de predadores (garapitos, renacuajos) y la cobertura de pasto. La productividad adulta (cantidad de hembras adultas que emergieron por metro cuadrado) no fue homogénea para todos los hábitats. La mayor productividad se encontró en los canales de irrigación (0,78 hembras/$m^2$) para *Anopheles* y en cámaras sépticas (2,86 hembras/$m^2$) para Culicinae y (0,86 hembras/$m^2$) para *Aedes*. Se encontró que *Anopheles arabiensis* era la especie *Anopheles* dominante. Este estudio documentó por primera vez la presencia de *An. funestus* en la zona central de Sudán.
**Conclusiones:** Se recomienda el mantenimiento de las tuberías con fugas y la adopción de irrigación intermitente para la gestión de fuentes de larvas, ya que estos hábitats estudiados representan la fuente principal que mantiene la población local de mosquitos durante la temporada seca de calor.

Translated from English version into Spanish by Maria Alejandra Aguada, through

TRANSLATORS
WITHOUT BORDERS
